# Synergism of primary and secondary interactions in a crystalline hydrogen peroxide complex with tin

**DOI:** 10.1038/s41467-024-50164-9

**Published:** 2024-07-09

**Authors:** Alexander G. Medvedev, Pavel A. Egorov, Alexey A. Mikhaylov, Evgeny S. Belyaev, Gayane A. Kirakosyan, Yulia G. Gorbunova, Oleg A. Filippov, Natalia V. Belkova, Elena S. Shubina, Maria N. Brekhovskikh, Anna A. Kirsanova, Maria V. Babak, Ovadia Lev, Petr V. Prikhodchenko

**Affiliations:** 1 Kurnakov Institute of General and Inorganic Chemistry, Russian Academy of Sciences, Moscow, Russian Federation; 2 Frumkin Institute of Physical Chemistry and Electrochemistry of the Russian Academy of Sciences, Moscow, Russian Federation; 3 Nesmeyanov Institute of Organoelement Compounds, Russian Academy of Sciences, Moscow, Russian Federation; 4 Drug Discovery Lab, Department of Chemistry, City University of Hong Kong, Kowloon, Hong Kong SAR China; 5 Casali Center of Applied Chemistry, Hebrew University of Jerusalem, Jerusalem, Israel

**Keywords:** Chemical bonding, Ligands, Metalloproteins

## Abstract

Despite the significance of H_2_O_2_-metal adducts in catalysis, materials science and biotechnology, the nature of the interactions between H_2_O_2_ and metal cations remains elusive and debatable. This is primarily due to the extremely weak coordinating ability of H_2_O_2_, which poses challenges in characterizing and understanding the specific nature of these interactions. Herein, we present an approach to obtain H_2_O_2_–metal complexes that employs neat H_2_O_2_ as both solvent and ligand. SnCl_4_ effectively binds H_2_O_2_, forming a SnCl_4_(H_2_O_2_)_2_ complex, as confirmed by ^119^Sn and ^17^O NMR spectroscopy. Crystalline adducts, SnCl_4_(H_2_O_2_)_2_·H_2_O_2_·18-crown-6 and 2[SnCl_4_(H_2_O_2_)(H_2_O)]·18-crown-6, are isolated and characterized by X-ray diffraction, providing the complete characterization of the hydrogen bonding of H_2_O_2_ ligands including geometric parameters and energy values. DFT analysis reveals the synergy between a coordinative bond of H_2_O_2_ with metal cation and its hydrogen bonding with a second coordination sphere. This synergism of primary and secondary interactions might be a key to understanding H_2_O_2_ reactivity in biological systems.

## Abstract (web summary)

Despite the significance of H2O2-metal adducts, the nature of the interactions between H2O2 and metal cations remains elusive due to the weak coordinating ability of H2O2, which poses challenges in characterizing the specific nature of these interactions. Herein, the authors obtain H2O2–metal complexes using H2O2 as both solvent and ligand.

## Introduction

Hydrogen peroxide, a highly stable reactive oxygen species^1^, is widely recognized for its vital contributions to diverse cellular functions including protection against oxidative stress, promotion of cellular differentiation, facilitation of cellular proliferation, and participation in redox signaling pathways^2–4^. Furthermore, owing to its oxidative properties, H_2_O_2_ holds significant importance in a range of industrial applications, including bleaching processes, wastewater treatment and various catalytic processes utilized in industry. While H_2_O_2_ can be employed in a metal-free processes the effectiveness and selectivity of H_2_O_2_ as an oxidant can be further enhanced through its kinetic activation by metal complexes^5,6^. For example, the activation of H_2_O_2_ by its coordination to heme enables the efficient utilization of H_2_O_2_ in various biochemical reactions catalyzed by cytochrome P450 heme-containing enzymes^7^. Despite the fundamental importance of these interactions, their molecular mechanism remains unclear due to the transient and labile nature of H_2_O_2_-metal adducts.

If we consider complexation as a reaction between Lewis acids and bases, the coordination ability of a ligand may be correlated with its basicity, which can be characterized in terms of its basicity constant (p*K*_b_, which is often substituted by the p*K*_a_ of the ligand’s corresponding conjugate acid) or proton affinity (PA; that is, the enthalpy of the B + H^+^ → BH^+^ reaction, where B = a base). As the PA of H_2_O_2_ is 4 kcal mol^−1^ less than that of H_2_O^8^, the coordination of H_2_O_2_ at low concentrations with a metal center is thermodynamically unfavorable in aqueous solutions. Indeed, Williams et al. reported that H_2_O_2_ complexes are thermodynamically unstable in aqueous solution unless the pH is sufficiently high to deprotonate H_2_O_2_ and thus favor the formation of hydroperoxo coordination compounds^9^. Subsequently, DiPasquale and Mayer demonstrated that H_2_O_2_ does not displace a very weakly bound perchlorate ligand from the gallium(III) center of a tetraphenylporphyrin complex^10^. Thus, as formulated by Mayer, H_2_O_2_ typically exhibits poor coordination ability due to its rather low PA, which is attributable to its electron-withdrawing hydroxyl (-OH) group being adjacent to an O atom^10^. A few examples of H_2_O_2_ coordinated with cobalt(II), nickel(II), and copper(II) in non-aqueous solutions were recently reported, supported by nuclear magnetic resonance (NMR) and cyclic voltammetry data^11,12^. In addition, many aqua complexes have been identified in solution and solid forms, but only one complex of H_2_O_2_ with a metal cation has been isolated and structurally characterized, namely a complex of H_2_O_2_ and zinc(II)^13^. However, the isomorphic substitution of H_2_O_2_ with H_2_O (in a 50:50 occupancy ratio) disordered the O atoms and tosyl fragments in this complex, preventing the positions of the H_2_O_2_ protons being determined objectively. In contrast to transition metals, p-block elements do not catalyze H_2_O_2_ decomposition. For example, tin compounds are known as H_2_O_2_ stabilizers as Sn^IV^ forms stable hydroperoxo complexes with high peroxide content^14^. However, to the best of our knowledge, the coordination of H_2_O_2_ with Sn^IV^ has not been reported.

Octahedral coordination is typical for Sn^IV^, but coordinatively unsaturated tin tetrachloride (SnCl_4_) is a strong Lewis acid and thus we hypothesized that it can bind H_2_O_2_ in the absence of ligands of higher basicity. We confirmed this hypothesis by performing ^119^Sn and ^17^O NMR studies that characterized H_2_O_2_ coordination by SnCl_4_ and H_2_O_2_ substitution by H_2_O, methanol (MeOH), and acetonitrile (MeCN) in the Sn^IV^ coordination sphere. In addition, we performed single-crystal X-ray diffraction (scXRD) analysis of two crystalline adducts of H_2_O_2_ with 18-crown-6, namely [SnCl_4_(H_2_O_2_)_2_]·H_2_O_2_·18-crown-6 (**1**) and 2[SnCl_4_(H_2_O_2_)(H_2_O)]·18-crown-6 (**2**), which enabled examination of the intermolecular interactions in these structures. Moreover, we performed density functional theory (DFT) modeling to unveil the synergy between various types of bonds in which H_2_O_2_ is engaged in **1** and **2** and how this effect stabilizes these complexes.

## Results and discussion

As H_2_O_2_ is less basic than other polar solvents^8^ and it does not form homogeneous solutions with non-coordinating solvents, we used neat H_2_O_2_ both as a ligand and as a solvent to study its interaction with SnCl_4_.

### ^119^Sn and ^17^O NMR studies

**Fig. 1:**
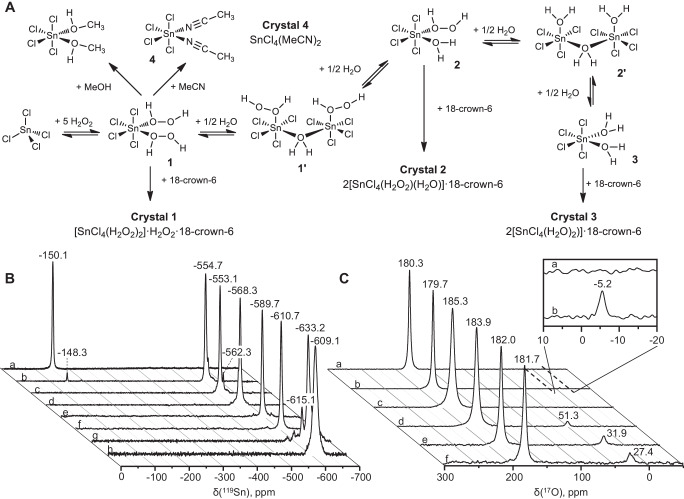
Complexation of tin tetrachloride in hydrogen peroxide solution supported by NMR spectroscopy. **A** Coordination of tin tetrachloride (SnCl_4_) with hydrogen peroxide (H_2_O_2_), formation of crystalline compounds **1**–**4** and assumed intermediate complexes supported by ^119^Sn and ^17^O NMR. **B** ^119^Sn nuclear magnetic resonance spectra of neat SnCl_4_ (a); an SnCl_4_–H_2_O_2_ (99.9%) system comprising a 1:5 molar ratio of SnCl_4_ to H_2_O_2_ (b); 3M SnCl_4_ in 99.9 wt.% H_2_O_2_ before (c) and after addition of 0.5 moles (d), 1 mole (e), 1.5 moles (f), and 3 moles of H_2_O with respect to Sn (g), and after addition of 3 moles of methanol with respect to Sn (h). **C** ^17^O NMR spectra of 99.9 wt.% H_2_O_2_ before (a) and after addition of 1 wt.% of water (H_2_O) (b); 3 M SnCl_4_ in 99.9 wt.% H_2_O_2_ before (c) and after addition of 0.5 moles (d), 1 mole (e), and 3 moles of H_2_O with respect to Sn (f).

The transformation of the Sn^IV^ coordination sphere upon addition of H_2_O_2_ proposed in Fig. 1A was studied by ^119^Sn and ^17^O NMR spectroscopy (Fig. 1B, C). The coordinatively unsaturated environment of Sn^IV^ in neat SnCl_4_ was revealed by its low-field signal in the ^119^Sn NMR spectrum (δ = −150 ppm; Fig. 1Ba). The ^17^O NMR spectrum of anhydrous H_2_O_2_ had a single signal at 180 ppm and no signals in the region of H_2_O, i.e., at approximately 0 ppm, confirming that it contained less than 0.5 wt.% H_2_O (Fig. 1Ca). The addition of 1 wt.% H_2_O resulted in the appearance of a signal in the ^17^O NMR spectrum at −5 ppm that was 0.8% of the integrated intensity of the H_2_O_2_ signal in this spectrum (Fig. 1Cb).

Careful addition of up to a fourfold molar excess of anhydrous H_2_O_2_ to SnCl_4_ yielded a biphasic mixture (Supplementary Movie 1), whereas addition of a fivefold molar excess of anhydrous H_2_O_2_ (1:1 v/v) to SnCl_4_ yielded a homogeneous mixture. ^119^Sn NMR spectroscopy of this SnCl_4_–5H_2_O_2_ system revealed a new high-field signal (δ_Sn_ = −554.7 ppm) assigned to a SnCl_4_(H_2_O_2_)_2_ complex **1** (Fig. 1A, 1Bb) and a low-intensity (3%) signal representing residual SnCl_4_. The latter signal was absent in the ^119^Sn NMR spectra of mixtures containing higher H_2_O_2_ concentrations (Fig. 1Bc). An H_2_O_2_ ligand in complex with tin contains two non-equivalent neighboring O atoms and thus its ^17^O NMR signals are quadrupole broadened and therefore not detectable. As such, only one signal was present in the ^17^O NMR spectrum (δ_O_ = 185.3 ppm) and was assigned to free H_2_O_2_ (Fig. 1Cc). In the ^119^Sn NMR spectra, a previously small signal at approximately −562 ppm became larger as the H_2_O_2_-to-SnCl_4_ ratio increased (to 9:1; Fig. 1Bc) and was assigned to the complex bearing a bridging aqua ligand [SnCl_4_(H_2_O_2_)]_2_(µ-H_2_O) (**1′**; Fig. 1A). Addition of H_2_O to give a 0.5 H_2_O-to-SnCl_4_ molar ratio resulted in [SnCl_4_(H_2_O_2_)]_2_(µ-H_2_O) being the dominant species (Fig. 1Bd), and its ^17^O NMR spectrum contained slightly upfield-shifted signals for free H_2_O_2_ (δ_O_ = 183.9 ppm) and a bridging aqua ligand signal (δ_O_ = 51.3 ppm; Fig. 1Cd). This conforms to ^119^Sn NMR being very sensitive to changes in both the first and second coordination sphere of Sn^IV^, so the subtle changes in the general composition of Sn^IV^ complexes cause shifts of corresponding signals in ^119^Sn NMR spectra.

Subsequent addition of another half equivalent of H_2_O resulted in the appearance of a high-field signal in ^119^Sn NMR (δ_Sn_ = −589.7 ppm; Fig. 1Be) and a new high-field H_2_O signal (δ_O_ = 31.9 ppm) in the corresponding ^17^O NMR spectrum (Fig. 1Ce) that we assigned to complex SnCl_4_(H_2_O_2_)(H_2_O) (**2**; Fig. 1A). This substantial change in the chemical shift of the O atom of a coordinated H_2_O suggested that it had changed from a bridging coordination mode to a terminal coordination mode. A gradual increase in the H_2_O concentration (to a threefold molar excess of H_2_O relative to SnCl_4_) led to the appearance of new high-field signals in the ^119^Sn NMR spectra, indicating the complete substitution of H_2_O_2_ ligands in the coordination sphere of Sn^IV^ in the original complex to form [SnCl_4_(H_2_O)]_2_(µ-H_2_O) (**2′**; δ_Sn_ = −610.7 ppm; Fig. 1Bf) and then the known^15^ SnCl_4_ diaqua complex SnCl_4_(H_2_O)_2_ (**3**; δ_Sn_ = −633.2 ppm; Fig. 1Bg). The ^17^O NMR spectrum of this compound exhibited the signals of an aqua ligand and H_2_O_2_ at δ_O_ = 27.4 and 181.7 ppm, respectively (Fig. 1Cf). The single resonance for the aqua ligand indicates a rapid exchange between the solvent and the ligand.

H_2_O_2_ ligands can also be substituted by other donor molecules. For example, the addition of a threefold molar excess (based on Sn) of MeOH to a SnCl_4_–H_2_O_2_ system resulted in the formation of SnCl_4_(MeOH)_2_, as confirmed by ^119^Sn NMR (δ_Sn_ = −609.1 ppm, Fig. 1Bh)^16^. Moreover, addition of MeCN to a solution of SnCl_4_ in anhydrous H_2_O_2_ resulted in the immediate formation of a crystalline complex SnCl_4_(MeCN)_2_ (**4**, Supplementary Fig. 4), as confirmed by scXRD^17^. This ease with which H_2_O_2_ ligands can be substituted is consistent with the large difference between the PA of H_2_O_2_ (161.2 kcal mol^−1^) and the PAs of H_2_O, MeOH, and MeCN (165.2, 180.3, and 186.2 kcal mol^−1^, respectively)^8^.

### Synthesis and crystal structure of SnCl_4_–H_2_O_2_ adducts with 18-crown-6

H_2_O_2_ always forms two hydrogen bonds, which stabilize crystalline adducts^18,19^. Similarly, an H_2_O_2_ ligand in a complex with zinc(II) was previously found to form hydrogen bonds with the proton-accepting tosyl groups of neighboring ligands^13^. Additionally, the formation of crystalline adducts of octahedral SnCl_4_(L)_2_ complexes bearing small ligands (e.g., diaqua and MeOH) and large organic molecules such as cyclodextrins, cucurbiturils, cryptands, and crown ethers was previously demonstrated^20^. In the current study, we examined whether SnCl_4_–H_2_O_2_ systems can be stabilized by 18-crown-6 ether, because this compound is impervious to oxidation and contains six oxygen atoms, which can act as proton acceptors. Moreover, hydrogen bonding of H_2_O_2_ with 18-crown-6 ether was previously revealed by scXRD analysis of a corresponding peroxosolvate^21^.

**Fig. 2:**
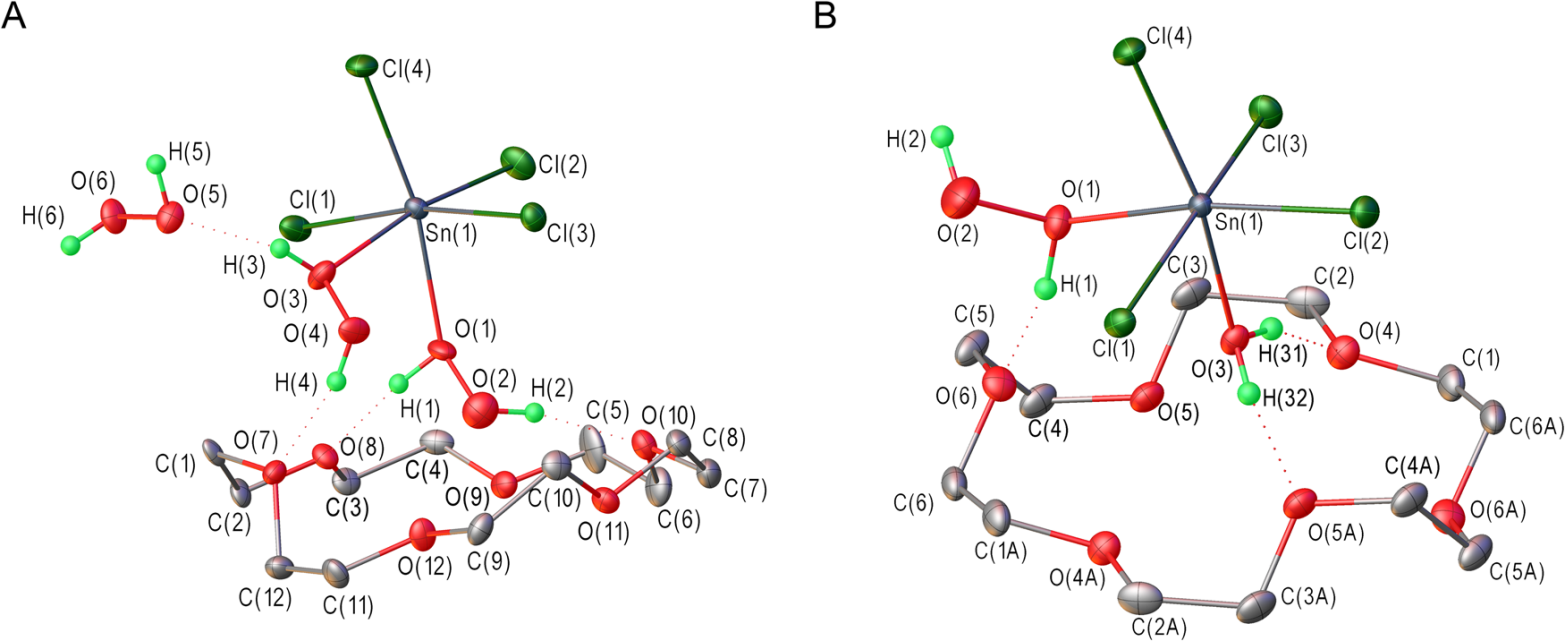
The crystal structures of hydrogen peroxide complexes with tin tetrachloride. **A** symmetric unit in **1**. **B** Asymmetric unit in **2**. 18-crown-6 molecule lies on crystallographic inversion center. Displacement ellipsoids are shown at a 50% probability level. Hydrogen bonds are represented by dotted lines. The H atoms of the macrocyclic ether are omitted for clarity.

Accordingly, crystals of **1–3** were obtained from solutions of SnCl_4_ in 99.9 wt.% H_2_O_2_ in the presence of 18-crown-6 with and without H_2_O, respectively, and analyzed by scXRD (Supplementary Table 1). This confirmed that **1–3** consisted of complexes with the compositions suggested by the NMR studies and unveiled a rich set of non-covalent interactions (Fig. 2 and Supplementary Fig. 1). In **1–3**, the Sn^IV^ atom is present in a distorted octahedral environment with four chlorine atoms and two O atoms of H_2_O_2_ or H_2_O molecules, resulting in a *cis* isomer with O–Sn–O angles significantly less than 90° (Fig. 2A,B, Supplementary Table 2). The distances between the Sn^IV^ and the O atoms of H_2_O_2_ (2.179(4) and 2.200(3) Å in **1**, and 2.225(3) Å in **2**) are much greater than those between the Sn^IV^ and the O atoms of H_2_O (2.138(3) Å in **2**, and 2.133(2), 2.138(2) Å in **3**) (Table 1 and Supplementary Table 2). This reflects the weaker coordination of H_2_O_2_ to Sn^IV^ than of H_2_O to Sn^IV^, as confirmed by addition of H_2_O resulting in the substitution of H_2_O_2_ by H_2_O. Moreover, the Sn–O distances in the complexes with aqua ligands exhibit a narrow range, but those in the complexes with H_2_O_2_ ligands exhibit a broader range. This disparity suggests that Sn–O interactions in the latter complexes are more significantly influenced by the strength of second-sphere hydrogen bonding than those in the aqua complexes. Thus, this hydrogen bonding fine-tunes the coordination of the H_2_O_2_ ligands.

**Table 1:** Selected geometric parameters obtained by scXRD analysis and DFT calculation (gas phase), and QTAIM-derived energetics for contacts involving the H_2_O_2_ ligand in 1 and 2 at the ωB97X-D3/TZVPP level of theory^a^

Cpd	d(Sn–O_2_H_2_), Å		*E*_Sn–O_,^b^ kcal mol^−1^	Contact	d(D…A), Å		d(H…A), Å		*E*_int_,^b^ kcal mol^−1^	Σ*E*_int_, kcal mol^−1^
	X-ray	DFT			X-ray	DFT	X-ray	DFT		
**1**	2.179(4)	2.261	16.4	O(1)H···O(8)	2.583(5)	2.642	1.74(3)	1.683	9.7	17.8
				O(2)H···O(10)	2.671(5)	2.733	1.82(3)	1.761	8.1	
	2.200(3)	2.323	13.4	O(3)H···O(5)	2.542(5)	2.690	1.74(3)	1.726	8.9	16.4
				O(4)H···O(7)^с^	2.730(5)	3.110	1.91(3)	2.419	2.0^d^	
				O(4)H···O(12)	3.156(5)	2.837	2.60(4)	1.945	5.5	
**2**	2.225(3)	2.358	12.3	O(1)H···O(6)	2.548(4)	2.631	1.70(3)	1.704	9.4	13.4
				O(2)H···Cl(3)^d^	3.114(4)	3.142	2.33(4)	2.267	4.0	

^a^See Fig. 2 for atom labeling.

^b^Calculated using Eq. 1 (see “Methods” section of the main text).

^c^O(7) and O(12) were neighboring oxygen atoms, and peroxide O(4)H switched its position between these O atoms in the DFT calculations.

^d^The intramolecular O(2)–H···Cl(4) hydrogen bond was identified in the calculated structure.

Interestingly, the H_2_O_2_ ligands in the structures of **1** and **2** do not form hydrogen bonds as proton acceptors. This is similar to the H_2_O_2_ hydrogen bonding in a previously reported^13^ Zn^II^ complex and may be caused by coordination to the Lewis-acidic Sn species. Instead, the H_2_O_2_ and H_2_O ligands participate as proton donors in hydrogen bonding with crown ether molecules (**1**–**3**), a chlorine atom in the adjacent SnCl_4_ fragment (**2**), and solvate H_2_O_2_ (**1**) (Supplementary Tables 3–5). The O(3)···O(5) distance in **1** (2.542(5) Å) and the O(1)···O(6) distance in **2** (2.548(4) Å) between the Sn-bound peroxo-OH moiety and adjacent H_2_O_2_ and ether oxygen, respectively, are much shorter than those in crystalline 18-crown-6 peroxosolvate (2.761(1)–3.040(1) Å)^21^. Furthermore, to the best of our knowledge, these distances are shorter than previously reported O···O distances in hydrogen bonds formed by H_2_O_2_ in crystalline peroxosolvates^20^. This suggests that the coordination of H_2_O_2_ with the Lewis acid (Sn^IV^) in **1** and **2** results in an increase in the acidity of H_2_O_2_ that makes it form short hydrogen bonds. The phenomenon of hydrogen-bond enhancement due to coordination with Lewis acids has been observed in various hydrogen-bond donors^22,23^ and widely applied in catalysis^24,25^. However, this phenomenon has not been reported to occur in complexes containing H_2_O_2_.

Binding to Sn leads to a shortening of the O–O bond in coordinated H_2_O_2_. Specifically, the O–O distances (1.422(5) and 1.443(5) Å in **1**, and 1.445(4) Å in **2**) are shorter than those in crystalline H_2_O_2_ (1.461(3) Å)^26^ and cesium hexahydroperoxo stannate (1.482(2) Å)^14^. In addition, the two O–O fragments of the H_2_O_2_ ligands in **1** are almost parallel to each other (Supplementary Fig. 5). However, remarkably, there is no hydrogen bond between the H_2_O_2_ ligands, despite these neighboring molecules bearing acidic protons. Moreover, the interligand O···O distances in **1** (O(1)···O(3) = 2.844(5) Å and O(2)···O(4) = 3.019(6) Å) are shorter than the sum of their van der Waals radii (3.04 Å), which could indicate the presence of a weak contact (e.g., a chalcogen bond).

### DFT calculations

To study the mutual influence of various types of bonding of H_2_O_2_ ligands we performed gas-phase DFT calculations for **1** and **2**. As mentioned above, the coordination of H_2_O_2_ with Sn^IV^ increases the acidity of H_2_O_2_, which increases the strength of the hydrogen bond that subsequently forms. According to DFT calculations, the sum of the energies of two hydrogen bonds formed by H_2_O_2_ molecules acting as proton donors correlates with the Sn–O distance, i.e., the high sum corresponds to the short distance, revealing the synergy between the coordination of an H_2_O_2_ ligand and its hydrogen bonding with the second coordination sphere (Table 1).

To highlight the synergy of primary (Sn–O) and secondary (hydrogen bond) interactions in an H_2_O_2_–Sn complex, we studied the interaction of an SnCl_4_(H_2_O_2_)_2_ complex with imidazole, which served as a model of proton-donating and proton-accepting molecules in the second coordination sphere. The imidazole moiety of histidine (His42) plays an important role in the peroxidase catalytic cycle^27^ contributing to the formation of the supposed iron-hydrogen peroxide complex with heme to give [Fe-OOH] form - a so-called Compound 0 - at the next step^27,28^. DFT calculations were performed for SnCl_4_(H_2_O_2_)_2_, H_2_O_2_·C_3_H_4_N_2_, SnCl_4_(H_2_O_2_)_2_·C_3_H_4_N_2_, and analogs containing the imidazolium cation (C_3_H_5_N_2_^+^), e.g., SnCl_4_(H_2_O_2_)_2_·C_3_H_5_N_2_^+^ (Table 2, Supplementary Fig. 6). The optimized structure of the SnCl_4_(H_2_O_2_)_2_·C_3_H_4_N_2_ adduct features the O–H···N hydrogen bond with an N···O distance of 2.622 Å, which correlates well with the corresponding interaction in the reported crystal structure of histidine peroxosolvate^29^. Adduct SnCl_4_(H_2_O_2_)_2_·C_3_H_5_N_2_^+^ contains an H_2_O_2_ ligand functioning as an acceptor of the acidic proton of the imidazolium cation, with an N···O distance of 2.967 Å.

**Table 2:** Bond distances (d, in Å) and energies (in kcal mol^−1^) for the hydrogen and coordination bonds in SnCl_4_(H_2_O_2_)_2_, H_2_O_2_·C_3_H_4_N_2_, SnCl_4_(H_2_O_2_)_2_·C_3_H_4_N_2_, H_2_O_2_·C_3_H_5_N_2_^+^, and SnCl_4_(H_2_O_2_)_2_·C_3_H_5_N_2_^+^ at the ωB97X-D3/TZVPP level of theory

	SnCl_4_(H_2_O_2_)_2_		H_2_O_2_·C_3_H_4_N_2_		SnCl_4_(H_2_O_2_)_2_·C_3_H_4_N_2_		
				
Δ*H*_*f*_^a^	−8.99 (−4.12*)		−6.36		−19.88 (−15.01*)		
Contact	d	*E*_int_^b^	d	*E*_int_	d	*E*_int_	ΔΔ*H*_coop_^с^
Sn–O1	2.371	11.7	–	–	2.284	15.1	−3.06
Sn–O3	2.334	13.1	–	–	2.339	12.9	
N···(H)O2	–	–	1.742	8.3	1.608	10.9	
			H_2_O_2_•C_3_H_5_N_2_^+^		SnCl_4_(H_2_O_2_)_2_•C_3_H_5_N_2_^+^		
					
Δ*H*_f_			−2.54		−11.52 (−6.66*)		
Contact	d	*E*_int_^b^	d	*E*_int_	d	*E*_int_	ΔΔ*H*_coop_
Sn–O1			*–*	–	2.432	9.9	1.06
Sn–O3			*–*	–	2.337	13.0	
N(H)···O2			1.837	7.0	1.988	4.9	

^*^ Energy relative to SnCl_4_(H_2_O_2_).

^a^Formation enthalpies, Δ*H*_f_, calculated relative to isolated reactants.

^b^Bond energies, *E*_int_, calculated using Eq. 1 (see “Methods” section of the main text).

^c^ΔΔ*H*_coop_, calculated using Eq. 2 (see “Methods” section of the main text).

In the SnCl_4_(H_2_O_2_)_2_·C_3_H_4_N_2_ adduct, the hydrogen bonding of coordinated H_2_O_2_ with imidazole leads to the shortening of the Sn–O distance for the hydrogen-bonded H_2_O_2_ ligand. The N···O distance of the hydrogen bond of the H_2_O_2_ ligand is also shorter than that in imidazole peroxosolvate, H_2_O_2_·C_3_H_4_N_2_. In contrast, the hydrogen bonding of peroxide oxygen with imidazolium in SnCl_4_(H_2_O_2_)_2_·C_3_H_5_N_2_^+^ causes a substantial elongation of the corresponding Sn–O bond. This reflects the mutual influence of primary (Sn–O) and secondary (hydrogen) bonds that is further supported by the energy analysis. The value and sign of the cooperative effect, ΔΔ*H*_coop_, for the SnCl_4_(H_2_O_2_)_2_ adduct with a second-coordination-sphere proton acceptor (imidazole molecule) or proton donor (imidazolium cation) estimated by Eq. 2 was used as a measure of the synergism or antagonism of primary and secondary interactions^30^.

A preliminary attempt to protonate SnCl_4_(H_2_O_2_)_2_ in gas-phase DFT calculations led to the dissociation of this complex. Therefore, we expected that the interaction of a proton donor with an H_2_O_2_ ligand would lead to a decrease in the stability of the complex. Indeed, hydrogen bonding of imidazolium to a distal oxygen has an antagonistic energetic effect (ΔΔ*H*_coop_ = 1.06 kcal mol^−1^). In contrast, a synergistic effect was found between H_2_O_2_ coordination and its proton donation to the imidazole fragment (ΔΔ*H*_coop_ = −3.06 kcal mol^−1^).

The distances between the Sn^IV^ coordination center and the ligand hydrogen-bond acceptor in the scXRD data of **1**–**3** and the calculated adduct SnCl_4_(H_2_O_2_)_2_•C_3_H_4_N_2_ are presented in Table 3. As expected, the distances between Sn^IV^ and the hydrogen-bond acceptor of the aqua ligand correlate with those for the proximal hydroxo group of the H_2_O_2_ ligand. However, the distance between the coordination center and the acceptor of the distal hydroxo group of the H_2_O_2_ ligand is always longer than that for the aqua ligand when the acceptor is of the same type. This observation also calls for a speculation on the coordination of H_2_O_2_ to enzymes’ heme, which occurs in aqueous systems. The Fe···N^His42^ distance in peroxidases is approximately 5.7 Å^31,32^, which is too long for activation of H_2_O but is suitable for activation of H_2_O_2_. In this hydrophobic pocket, the OH···N hydrogen bonding of the distal OH group to His42 should stabilize the binding of H_2_O_2_ to a heme Fe. One can speculate that due to this unique hydrogen bond of the distal proton, the enzyme can differentiate between Fe·OH_2_ and Fe·O_2_H_2_ complexes, as the cooperative effect (as estimated herein; Table 2) would overcome a stronger Fe–O bond with H_2_O than with H_2_O_2_ and stabilize the encounter [Fe–O_2_H_2_] complex. Furthermore, as the coordination to an Fe ion increases the acidity of the proximal OH group, it should facilitate deprotonation of Compound 0 yielding [Fe–OOH] hydroperoxo complex.

**Table 3:** Distances between each Sn^IV^ coordination center and its ligand’s hydrogen-bond acceptor

Compound	Method	d(Sn···A) (A = O, Cl, or N), Å		
		H_2_O_2_		H_2_O
		Proximal	Distal	
[SnCl_4_(H_2_O_2_)_2_]·H_2_O_2_·18-crown-6 (**1**)	scXRD	4.217 (O) 4.274 (O)	4.867 (O) 4.548 (O)	–
2[SnCl_4_(H_2_O_2_)(H_2_O)]·18-crown-6 (**2**)	scXRD	4.281 (O)	5.315 (Cl)	4.031 (O) 4.339 (O)
2[SnCl_4_(H_2_O)_2_]·18-crown-6 (**3**)	scXRD	–	–	4.011 (O) 4.415 (O) 4.187 (O) 4.664 (Cl)
SnCl_4_(H_2_O_2_)_2_·C_3_H_4_N_2_	DFT	–	4.695 (N)	–

The scarcity of structurally resolved H_2_O_2_ complexes has hindered an examination of the structure and bonding of H_2_O_2_ ligands in reaction intermediates and life-sustaining biocomplexes. This limitation is attributable to H_2_O_2_ being less basic than the common coordinating solvents by which it is replaced in a metal coordination sphere. In addition, H_2_O_2_ is poorly soluble in most non-coordinating solvents. In this study, we suggested a synthetic approach based on the use of pure H_2_O_2_ as both solvent and ligand. Thus, coordinatively unsaturated SnCl_4_ effectively binds H_2_O_2_ yielding SnCl_4_(H_2_O_2_)_2_, which was characterized by ^119^Sn and ^17^O NMR spectroscopy. This complex comprises rather strong Sn-O bonds (12–16 kcal mol^−1^ according to DFT analysis), but its H_2_O_2_ ligands could be easily substituted in the Sn^IV^ coordination sphere by molecules of higher basicity, i.e., MeOH, MeCN, and even H_2_O. The use of 18-crown-6 as a bulky yet stable H_2_O_2_-proton acceptor stabilized SnCl_4_(H_2_O_2_)_2_ as its 18-crown-6 adduct (**1**). The addition of H_2_O gave the stepwise substitution products **2** and **3**. ScXRD analysis of these complexes revealed their rich set of non-covalent interactions, including the shortest O(H)···O distances in hydrogen bonds formed by H_2_O_2_ in known crystal structures. Complemented by the results of our DFT analysis, this demonstrates the synergistic effects of a coordination bond with Sn^IV^ and hydrogen bonding with a second coordination sphere on the properties of an H_2_O_2_ ligand. The energies of two hydrogen bonds formed by each H_2_O_2_ ligand acting as a proton donor correlate with the Sn–O distance in **1** and **2**, with the higher hydrogen-bond energy value corresponding to the shorter Sn–O distance. Remarkably, none of the H_2_O_2_ ligands participated in hydrogen bonding as proton acceptors, despite the proximity of acidic protons. Our DFT study of model SnCl_4_(H_2_O_2_)_2_ adducts with imidazole/imidazolium suggested that this is due to the antagonistic energetic effect of such interactions.

In summary, this study demonstrated that second-coordination-sphere hydrogen bonding plays a key role in the stabilization of H_2_O_2_ coordination. The non-covalent interactions of H_2_O_2_ ligands not only contribute to the total energy of the system but also increase the basicity of the H_2_O_2_ ligand, which enhances coordination bonding. This explains why H_2_O_2_ coordination, despite being impossible in aqueous solution under equilibrium conditions, is common in nature, such as in oxygenases. Coordination with a Lewis acid has previously been proposed to be a key factor in the activation of H_2_O_2_ for the oxidation of organic substrates^5^. Therefore, we envisage prospects for the development of new catalytic systems in which the distance between the coordination center and the hydrogen bond acceptor is approximately 5 Å. This would make it possible to utilize the synergism of the primary and secondary interactions and ensure the coordination of H_2_O_2_ in the presence of H_2_O or other polar molecules.

## Methods

### Synthesis of anhydrous H_2_O_2_ and SnCl_4_

Caution! Working with concentrated H_2_O_2_ and chlorine is hazardous and requires appropriate precautions to be taken.

Small amounts of anhydrous H_2_O_2_ can be obtained from its crystalline adducts with organic compounds^33,34^. However, this requires the use of organic solvents (diethyl ether or MeCN) that may absorb H_2_O and other impurities, and also may remain in the product H_2_O_2_ and thus interact with SnCl_4_ in the next step. Therefore, in the current study, we purified commercial H_2_O_2_ via a two-stage vacuum distillation process. First, 30 wt.% H_2_O_2_ was distilled under vacuum to remove stabilizers and other impurities and afford 18 wt.% pure aqueous H_2_O_2_. Second, this H_2_O_2_ solution was concentrated by rectification under vacuum, controlling the boiling by passing argon (Ar), to afford 99.9 wt.% H_2_O_2_ (as determined by permanganometry; Supplementary Methods).

As commercial SnCl_4_ may contain impurities that can catalyze H_2_O_2_ decomposition, we synthesized SnCl_4_ from ultrapure metal Sn by chlorination followed by rectification (Supplementary Methods).

### NMR spectroscopy

The solutions for NMR experiments were prepared in an Ar-filled glovebox (O_2_ and H_2_O concentrations < 0.1 ppm) and then immediately placed in the spectrometer (Supplementary Methods). The time between the preparation of the solutions and the NMR experiments did not exceed 10 min. ^17^O and ^119^Sn NMR spectra (δ, ppm) were collected at 303 K on a Bruker AVANCE III 600 spectrometer operating at 81.36 MHz and 223.79 MHz, respectively. ^17^O and ^119^Sn chemical shifts were referenced to H_2_O and tetramethyltin, respectively. NMR spectra were processed using TopSpin software.

### ScXRD

Single crystals of **1–4** that were suitable for X-ray analysis were collected from the corresponding mother liquors without additional recrystallization, placed on microscope slides, and then coated with a perfluorinated oil (Fomblin YR-1800). Subsequently, appropriate single crystals were mounted on MicroMeshes™ (MiTeGen) and then immediately positioned beneath a cold stream of nitrogen on the diffractometer, which was a Bruker D8 Venture instrument that used graphite monochromatized molybdenum K-alpha radiation (λ = 0.71073 Å) and was operated in ω-scan mode at 100 K. Absorption corrections based on measurements of equivalent reflections were applied^35^. The structures were solved by direct methods and refined by full matrix least-squares on F^2^ with anisotropic thermal parameters for all non-hydrogen atoms^36^. The hydrogen atoms of H_2_O_2_ and H_2_O molecules in **1**–**3** were found from difference Fourier synthesis and refined with distance restraints. The hydrogen atoms of 18-crown-6 in **1**–**3** and MeCN solvent in **4** were placed in idealized positions and refined using a riding model. Additional crystallographic data for **1**–**4** are provided in the Supplementary Information (Supplementary Figs. 1–4, Supplementary Tables 1–5).

ScXRD of **1** indicated a relatively short distance between H(4) and O(12), i.e., 2.60(4) Å. However, it was much longer than a typical O–H···O hydrogen bond, and its O–H···O angle (124.7°) lay outside the normal range. Thus, this contact was not attributable to a hydrogen bond and appeared to be a forced contact due to crystal packing.

The X-ray structure of **4** exhibited better resolution (see Supplementary Table 1) than that of a previously reported structure^17^.

### DFT calculations

Clusters containing the fragments of asymmetric units of **1** and **2** were taken from the corresponding scXRD data, and calculations were performed using various approaches (Supplementary Methods, Supplementary Data 1). Optimization at the ωB97X-D3/TZVPP level of theory afforded the best correlation between the calculated Sn–O distances and those obtained from scXRD (Table 1) and is therefore used in the discussion of the results below. The quantum theory of atoms in molecules was applied to analyze the electron density parameters at the O–H···O hydrogen-bond critical points (Table 1 and Supplementary Table 6).

In these calculations, H_2_O was used as a solvent (in the conductor-like polarizable continuum model approach) because it closely approximates the solvent properties of H_2_O_2_ (i.e., its dielectric constant and acidity/basicity) and because polar solvents typically weaken non-covalent interactions, meaning that the detection of a pronounced effect in such a solvent serves as strong evidence of the proposed concept.

The energies of non-covalent interactions in the optimized clusters of **1** and **2** were estimated according to Espinosa’s approach^37^ (Eq. 1) and are presented in Table 1.

1 $${{{E}}}_{{{{{\mathrm{int}}}}}}[{{{{{\rm{kcal\; }}}}}}{{{{{{\rm{mol}}}}}}}^{-1}]=269.2{G}_{b}[{{{{{\rm{atomic\; units}}}}}}]$$

where *G*_*b*_ is a Lagrangian of kinetic energy density at the bond critical point.

The cooperative effect, ΔΔ*H*_coop_, was calculated according to ref. ^30^:

2 $${\Delta \Delta H}_{{{{{{\rm{coop}}}}}}}({{{{{\rm{ABC}}}}}})={H}_{{{{{{\rm{ABC}}}}}}}-({H}_{{{{{{\rm{AB}}}}}}}+{H}_{{{{{{\rm{BC}}}}}}}+{H}_{{{{{{\rm{AC}}}}}}})+({H}_{{{{{{\rm{A}}}}}}}+{H}_{{{{{{\rm{B}}}}}}}+{H}_{{{{{{\rm{C}}}}}}})$$

where *H* are enthalpies of the corresponding trimer (SnCl_4_(H_2_O_2_)_2_·C_3_H_4+n_N_2_^n+^), dimers (SnCl_4_(H_2_O_2_)_2_, H_2_O_2_·C_3_H_4+n_N_2_^n+^ and SnCl_4_(H_2_O_2_)//C_3_H_4+n_N_2_^n+^) and monomers (SnCl_4_(H_2_O_2_), H_2_O_2_ and C_3_H_4+n_N_2_^n+^) at the trimer geometry; n = 0 for imidazole, and n = 1 for imidazolium.

### Supplementary information

Supplementary Information

Peer Review File

Description of Additional Supplementary Files

Supplementary Data 1

Supplementary Movie 1

## Data Availability

The X-Ray crystallographic data for the structures reported in this Article have been deposited at the Cambridge Crystallographic Data Centre (CCDC) under deposition numbers CCDC 2260843 (**1**), 2260844 (**2**), 2260845 (**3**) and 2260846 (**4**). These data can be obtained free of charge via https://www.ccdc.cam.ac.uk/structures/. The equilibrium Cartesian coordinates data generated in this study are provided as the Supplementary Data 1. All data are available in the main text, the Supplementary Information and from the corresponding authors.
