# Supplementary material for: Synergism of primary and secondary interactions in a crystalline hydrogen peroxide complex with tin
Source: Nat Commun. 2024 Jul 9;15:5758. doi: 10.1038/s41467-024-50164-9 (PMC11233698; doi:10.1038/s41467-024-50164-9)
Supplement: Supplementary file 1 — Supplementary Information [file 41467_2024_50164_MOESM1_ESM.pdf]

Supplementary Information

Synergism of primary and secondary interactions in a crystalline hydrogen peroxide complex with tin

Alexander G. Medvedev[1], Pavel A. Egorov[1], Alexey A. Mikhaylov[1], Evgeny S. Belyaev[2], Gayane A. Kirakosyan[1,2], Yulia G. Gorbunova[1,2], Oleg A. Filippov[3], Natalia V. Belkova[3], Elena S. Shubina[3], Maria N. Brekhovskikh[1], Anna A. Kirsanova[4], Maria V. Babak[4]*, Ovadia Lev[5]* & Petr V. Prikhodchenko[1]*

[1] Kurnakov Institute of General and Inorganic Chemistry, Russian Academy of Sciences, Leninskii pr. 31, Moscow 119991, Russian Federation

[2] Frumkin Institute of Physical Chemistry and Electrochemistry of the Russian Academy of Sciences, Leninskii pr. 31-4, Moscow 119071, Russian Federation

[3] Nesmeyanov Institute of Organoelement Compounds, Russian Academy of Sciences, Vavilov Str. 28, Moscow 119334, Russian Federation

[4] Drug Discovery Lab, Department of Chemistry, City University of Hong Kong, Kowloon, Hong Kong SAR 999077, China

[5] Casali Center of Applied Chemistry, Hebrew University of Jerusalem, Jerusalem 9190401, Israel

* Corresponding Authors: ovadia@mail.huji.ac.il, mbabak@cityu.edu.hk, prikhman@gmail.com

1. Supplementary Methods S3
   1.1 General Information S3
   1.2 Preparation of solutions for NMR studies S3
   1.3 Synthesis.. S4
   1.4 Characterization S5
   1.5 Calculations.. S5

Supplementary Fig. 1. ORTEP representation of the asymmetric unit of 1. Displacement ellipsoids are shown at 50% probability level. S6

Supplementary Fig. 2. ORTEP representation of the asymmetric unit of 2. Displacement ellipsoids are shown at 50% probability level. S7

Supplementary Fig. 3. ORTEP representation of the asymmetric unit of 3. Displacement ellipsoids are shown at 50% probability level. S8

Supplementary Fig. 4. ORTEP representation of the asymmetric unit of 4. Displacement ellipsoids are shown at 50% probability level. S9

Supplementary Fig. 5. The O…O distances in $SnCl_4(H_2O_2)_2{\cdot}H_2O_2$ fragment in 1. Crown ether is omitted for clarity........ S9

Supplementary Fig. 6. The optimized structures $SnCl_4(H_2O_2)_2$ (a), $H_2O_2{\cdot}C_3H_4N_2$ (b), $SnCl_4(H_2O_2)_2{\cdot}C_3H_4N_2$ (c), $H_2O_2{\cdot}C_3H_5N_2^+$ (d) and $SnCl_4(H_2O_2)_2{\cdot}C_3H_5N_2^+$ (e) at the ωB97X-D3/TZVPP level of theory........ S10-S12

Supplementary Fig. 7. Experimental (a) and theoretical (b) powder XRD diffractograms of compound 3........ S13

Supplementary Fig. 8. The FTIR spectrum of compound 3. .... S13

Supplementary Fig. 9. Experimental (a) and theoretical (b) powder XRD diffractograms of compound 4........ S13

Supplementary Table 1. Crystal data and details of X-ray analysis of 1-4........ S14

Supplementary Table 2. Selected geometric parameters of 1-3. .... S15

Supplementary Table 3. Geometric parameters of hydrogen bonds in the structure 1........ S15

Supplementary Table 4. Geometric parameters of hydrogen bonds in the structure 2........ S15

Supplementary Table 5. Geometric parameters of hydrogen bonds in the structure 3........ S16

Supplementary Table 6. Selected properties of the electron density at bond critical points according to Bader analysis. S16

Supplementary Table 7. FTIR band assignment of 3. .... S17

Supplementary References........ S18

1. Supplementary Methods

1.1 General Information

Tin (99.999 wt.%), chlorine (99.9 wt.%), hydrogen peroxide (30 wt.%), 18-crown-6 ether (99 wt.%), methanol, acetonitrile and sodium sulphate were purchased from Sigma-Aldrich. Solvents were dried over molecular sieves (3 Å or 4 Å) and freshly distilled prior use.

Glassware for peroxide rich solutions: All glassware were treated by filling with 1M NaOH for 1 day, then with 1M nitric acid for an additional day, and finally with 10 wt.% hydrogen peroxide for an further day. Dichromate or permanganate treatment should be avoided.

Safety note: Concentrating hydrogen peroxide solutions and working with them require safety precautions. Handling procedures for concentrated hydrogen peroxide are described in detail (danger of explosion!).[1]

1.2 Preparation of solutions for NMR studies

The solutions for NMR experiments were prepared in an argon filled glovebox (O_2 and H_2O content less than 0.1 ppm) and immediately placed to the spectrometer. The time between the preparation of the solution and the NMR experiment did not exceed 5-10 min. The approximate molar concentration of the studied solutions was estimated based on the assumption that the resulting volume is equal to the sum of the volumes of the liquid components (tin tetrachloride, hydrogen peroxide, water, methanol, acetonitrile).

Solution 1. 0.7 ml of $SnCl_4$.

Solution 2. 99.9 wt.% H_2O_2 (0.353 mL, 0.512 g, 15.0 mmol) was added dropwise to $SnCl_4$ (0.352 mL, 0.784 g, 3.0 mmol).

Solution 3. 99.9 wt.% H_2O_2 (0.452 mL, 0.655 g, 19.2 mmol) was added dropwise to $SnCl_4$ (0.245 mL, 0.545 g, 2.1 mmol).

Solution 4. 99.9 wt.% H_2O_2 (0.190 mL, 0.276 g, 8.1 mmol) was mixed with 95.0 wt.% H_2O_2 (0.265 mL, 0.376 g, 10.5 mmol) and added dropwise to $SnCl_4$ (0.245 mL, 0.545 g, 2.1 mmol) resulting in $[H_2O]/[Sn]=0.5/1$ molar ratio.

Solution 5. 99.9 wt.% H_2O_2 (0.182 mL, 0.264 g, 7.8 mmol) was mixed with 90.0 wt.% H_2O_2 (0.273 mL, 0.380 g, 10.1 mmol) and added dropwise to $SnCl_4$ (0.245 mL, 0.545 g, 2.1 mmol) resulting in $[H_2O]/[Sn]=1/1$ molar ratio.

Solution 6. 99.9 wt.% H_2O_2 (0.048 mL, 0.070 g, 2.0 mmol) was mixed with 90.0 wt.% H_2O_2 (0.407 mL, 0.567 g, 15.0 mmol) and added dropwise to $SnCl_4$ (0.245 mL, 0.545 g, 2.1 mmol) resulting in $[H_2O]/[Sn]=1.5/1$ molar ratio.

Solution 7. 99.9 wt.% H_2O_2 (0.033 mL, 0.048 g, 1.4 mmol) was mixed with 80.0 wt.% H_2O_2 (0.422 mL, 0.566 g, 13.3 mmol) and added dropwise to $SnCl_4$ (0.245 mL, 0.545 g, 2.1 mmol) resulting in $[H_2O]/[Sn]=3/1$ molar ratio.

Solution 8. 99.9 wt.% H_2O_2 (0.452 mL, 0.655 g, 19.2 mmol) was added dropwise to $SnCl_4$ (0.245mL, 0.545 g, 2.1 mmol, *solution 3*). Methanol (0.254 mL, 0.201 g, 6.3 mmol) was added to the 0.7 ml of *solution 3*.

1.3 Synthesis

Purification of H_2O_2.

Commercial hydrogen peroxide was concentrated by vacuum distillation. On the first stage, 30 wt.% hydrogen peroxide (45 mL, 50 g) was distilled under vacuum to remove stabilizers and other impurities. Thus obtained 18 wt.% pure aqueous hydrogen peroxide (35 mL, 37 g) was concentrated by rectification under vacuum and controlled boiling by passing argon resulted in 99.9 wt.% hydrogen peroxide (3.45 ml, 5 g) as determined by permanganometry.[1]

Synthesis and purification of $SnCl_4$ (5N, 99.999 wt.%)

Tin tetrachloride was prepared by reaction of Sn with chlorine gas in a quartz installation that consisted of a distilling column (reflux condenser), still, quartz hose, and receiver for the sampling of prepared tin tetrachloride. Deep purification of tin tetrachloride was conducted in a perforated-plate rectification column (high-purity grade quartz).

*Synthesis of $[SnCl_4(H_2O_2)_2]\cdot H_2O_2\cdot 18$-crown-6 (**1**)*

Solid 18-crown-6 ether (100 mg, 0.38 mmol) was carefully introduced to the 3M tin tetrachloride solution in 99.9 wt.% hydrogen peroxide (*solution 3*, 0.126 mL, containing 0.38 mmol of $SnCl_4$). Immediate precipitation of product was observed. The precipitate was transferred to the perfluorinated oil on the glass slide. All manipulations were performed in an argon filled glovebox.

*Synthesis of $2[SnCl_4(H_2O_2)(H_2O)]\cdot 18$-crown-6 (**2**)*

Solid 18-crown-6 ether (100 mg, 0.38 mmol) was carefully introduced to the 0.126 mL of *solution 5* (containing 0.38 mmol of $SnCl_4$). Immediate precipitation of product was observed. The precipitate was transferred to the perfluorinated oil on the glass slide. All manipulations were performed in an argon filled glovebox.

*Synthesis of $2[SnCl_4(H_2O)_2]\cdot 18$-crown-6 (**3**)*

Solid 18-crown-6 ether (100 mg, 0.38 mmol) was carefully introduced to the 0.126 mL of *solution 7* (containing 0.38 mmol of $SnCl_4$). Immediate precipitation of product was observed. This protocol was used to obtain single crystals for scXRD.

The above protocol was optimized in accordance with the composition of product **3** as determined from X-ray diffraction analysis with a reduction in the amount of 18-crown-6 ether by a factor of two. Solid 18-crown-6 ether (50 mg, 0.19 mmol) was carefully introduced to 0.126 mL of *solution 7* (containing 0.38 mmol of $SnCl_4$). Immediate precipitation of product was observed. Sample was washed by 10 mL of diethyl ether and dried. All manipulations were performed in an argon filled glovebox. Yield by Sn 73.7% (120 mg).
The product was confirmed by chemical analysis (tin, carbon and hydrogen content), X-ray powder diffraction and FTIR spectroscopy.
Anal. Calc. for $C_{12}H_{32}Cl_8O_{10}Sn_2$ (**3**): Sn, 27.69; C, 16.81; H, 3.76. Found: Sn, 27.32; C, 16.74; H, 3.91.
The experimental powder X-ray diffractogram of **3** conforms to the diffractogram simulated from the single crystal X-ray diffraction data (Supplementary Fig. 7).

The FTIR spectrum of compound **3** (Supplementary Fig. 8) is similar to the previously published for [*trans*-$SnCl_4(H_2O)_2$]·18-crown-6.[2] The assignments of the FTIR bands are presented in Supplementary Table 7.

*Synthesis of $SnCl_4(CH_3CN)_2$ (**4**)*

Acetonitrile (0.330 mL, 6.3 mmol) was added to the 0.7 ml of *solution 3*. Immediate precipitate formation was observed. Crystals of **4** were filtered on a glass filter and transferred into low background quartz XRD sample holder, and then coated with a perfluorinated oil (Fomblin YR-1800). All manipulations were performed in an argon filled glovebox. The experimental powder X-ray diffractogram of **4** fitted with the diffractogram simulated from the single crystal X-ray diffraction data (Supplementary Fig. 9). The small low-angle shift of the experimental reflections relative to the simulated diffraction pattern was attributed to displacement of the wet sample due to shrinkage during measurement.

1.4 Characterization

^{17}O, ^{119}Sn NMR spectra δ, ppm were measured at 303K on a Bruker AVANCE III 600 spectrometer (Bruker, Germany) operating at 81.36 MHz and 223.79 MHz, respectively. ^{17}O and ^{119}Sn chemical shifts were referenced to H_2O and Me_4Sn, respectively. NMR spectra were processed by TopSpin software.

Elemental analysis. Carbon and hydrogen content were determined using the vario MICRO cube analyzer (Elementar, Germany). Tin content was determined by gravimetric analysis as tin dioxide.[3]

Infrared spectroscopy. Fourier-transform infrared spectroscopy (FTIR) spectra were recorded on a JASCO FT/IR-4600 spectrometer (Jasco, Japan) equipped with ATR PRO ONE Single-reflection ATR accessory.

X-ray powder diffraction measurements were performed on a D8 Advance diffractometer (Bruker AXS, Germany) with a goniometer radius of 280 mm. The powder samples were filled into low background quartz sample holders. XRD patterns in the range 5° to 30° 2θ were recorded at room temperature using CuKα radiation (λ=1.5418 Å) under the following measurement conditions: tube voltage of 40 kV, tube current of 40 mA, step scan mode with a step size 0.02° 2θ, and counting time of 1s/step. XRD patterns were processed by DiffracPlus software.

1.5 Calculations

For geometry optimization of the complexes ORCA v5.0.4 was used.[4,5] ZORA Hamiltonian[6] was used for treating of relativistic effects, ωB97X functional[7] with Grimme's DFT-D3 correction[8] was applied. The ZORA-TZVPP for light (H, C, N, O, Cl) atoms, and scalar all-electron relativistic TZVPP basis set (SARC-ZORA-TZVPP)[9] for Sn atom were used with the general-purpose auxiliary basis set SARC/J. Implicit solvation (water, ε=80.4) were introduced by CPCM model.[10]

Additionally same complexes were optimized with Gaussian09[11] with ωB97XD,[12] B3LYP[13,14] and PBE0[15] functionals with the def2-TZVPP basis set,[16] supplied with the ECP for Sn atom.[17] Optimizations were done in the gas phase, benzene (ε=2.2706) and water (ε=78.3553). The equilibrium Cartesian coordinates of atoms in **1**, **2,** $SnCl_4$, H_2O_2, $C_3H_4N_2$, $C_3H_5N_2^+$, $SnCl_4(H_2O_2)$, $SnCl_4(H_2O_2)_2$, $H_2O_2·C_3H_4N_2$, $SnCl_4(H_2O_2)_2·C_3H_4N_2$, $H_2O_2·C_3H_5N_2^+$, $SnCl_4(H_2O_2)_2·C_3H_5N_2^+$ complexes are presented as Supplementary Data.

Bader analysis[18] of electron density was conducted using AIMALL software.[19]

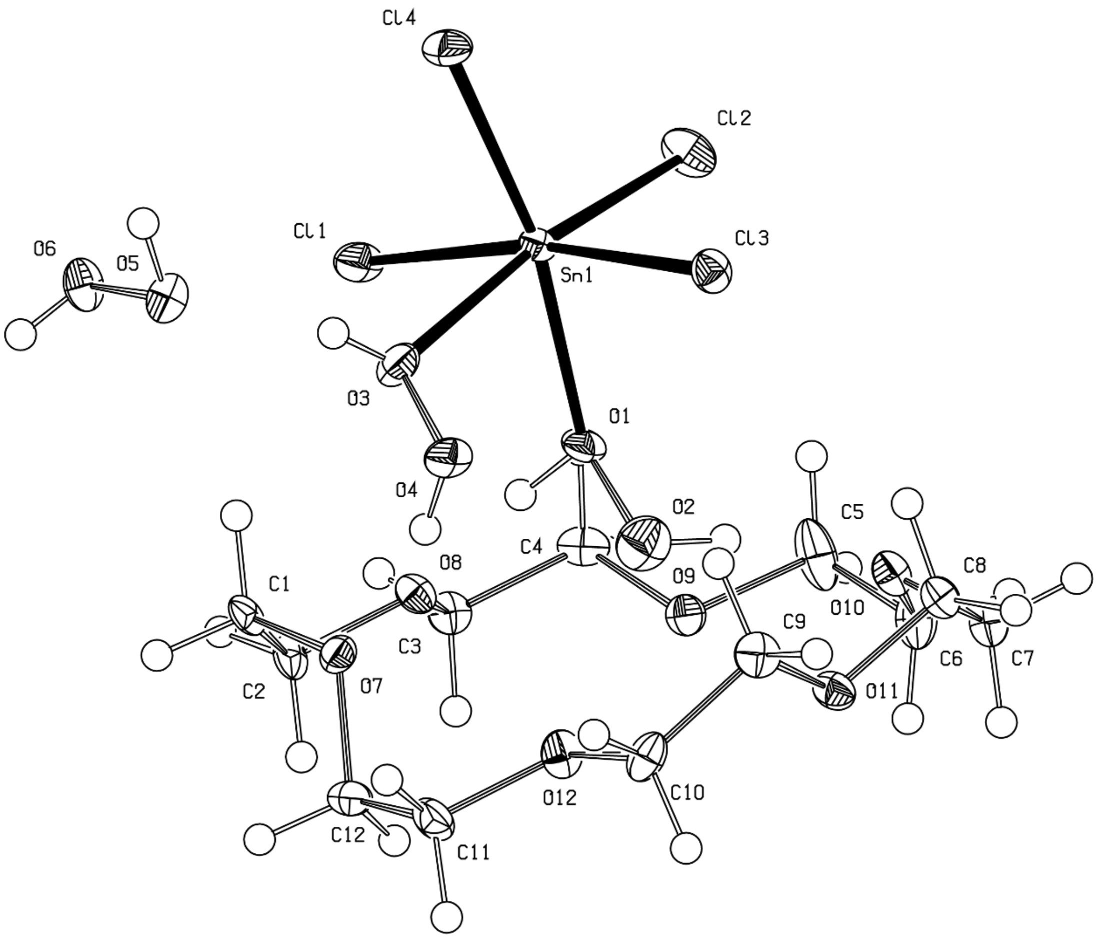

Supplementary Fig. 1. ORTEP representation of the asymmetric unit of **1**. Displacement ellipsoids are shown at 50% probability level.

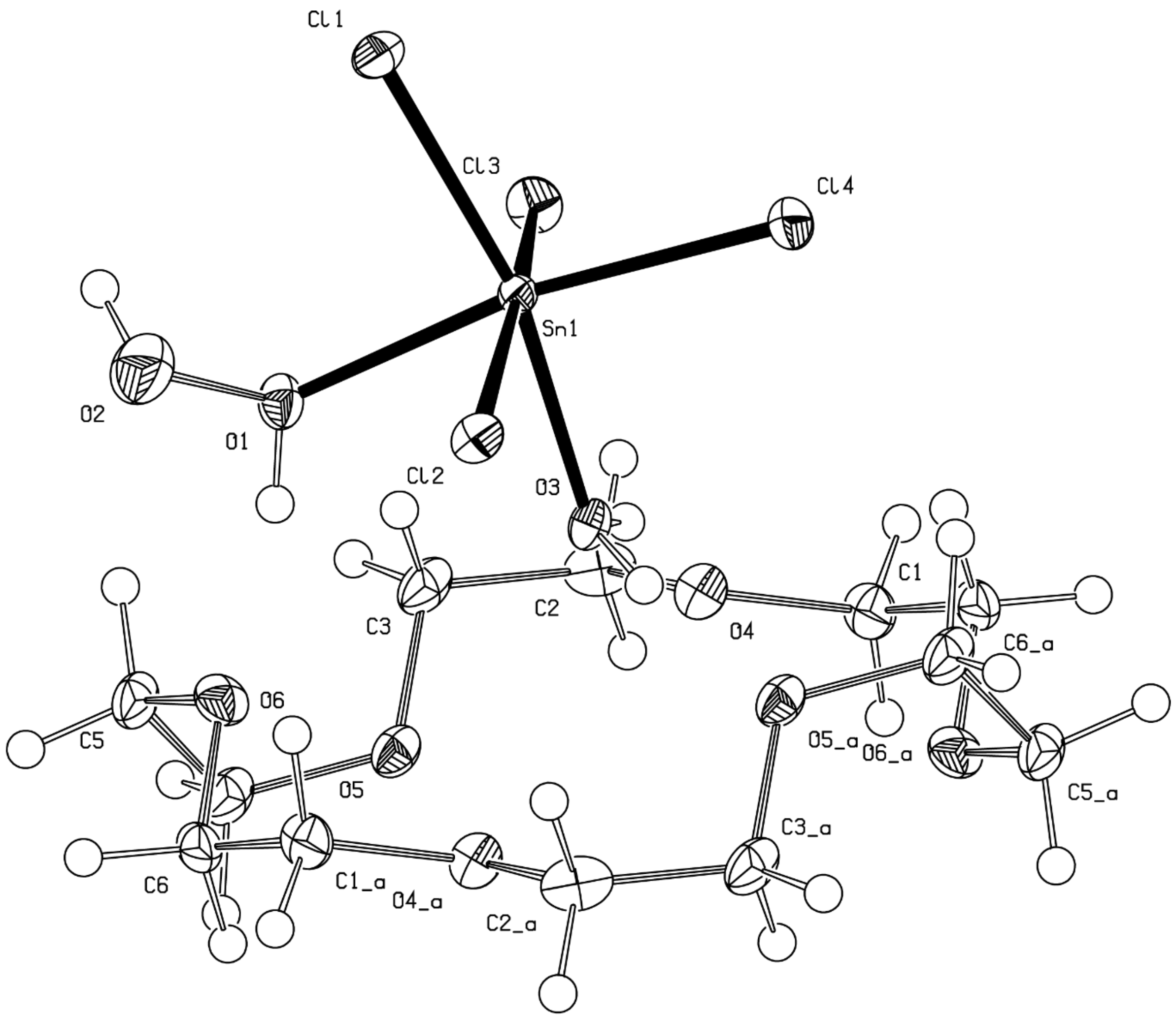

Supplementary Fig. 2. ORTEP representation of the asymmetric unit of **2**. Displacement ellipsoids are shown at 50% probability level.

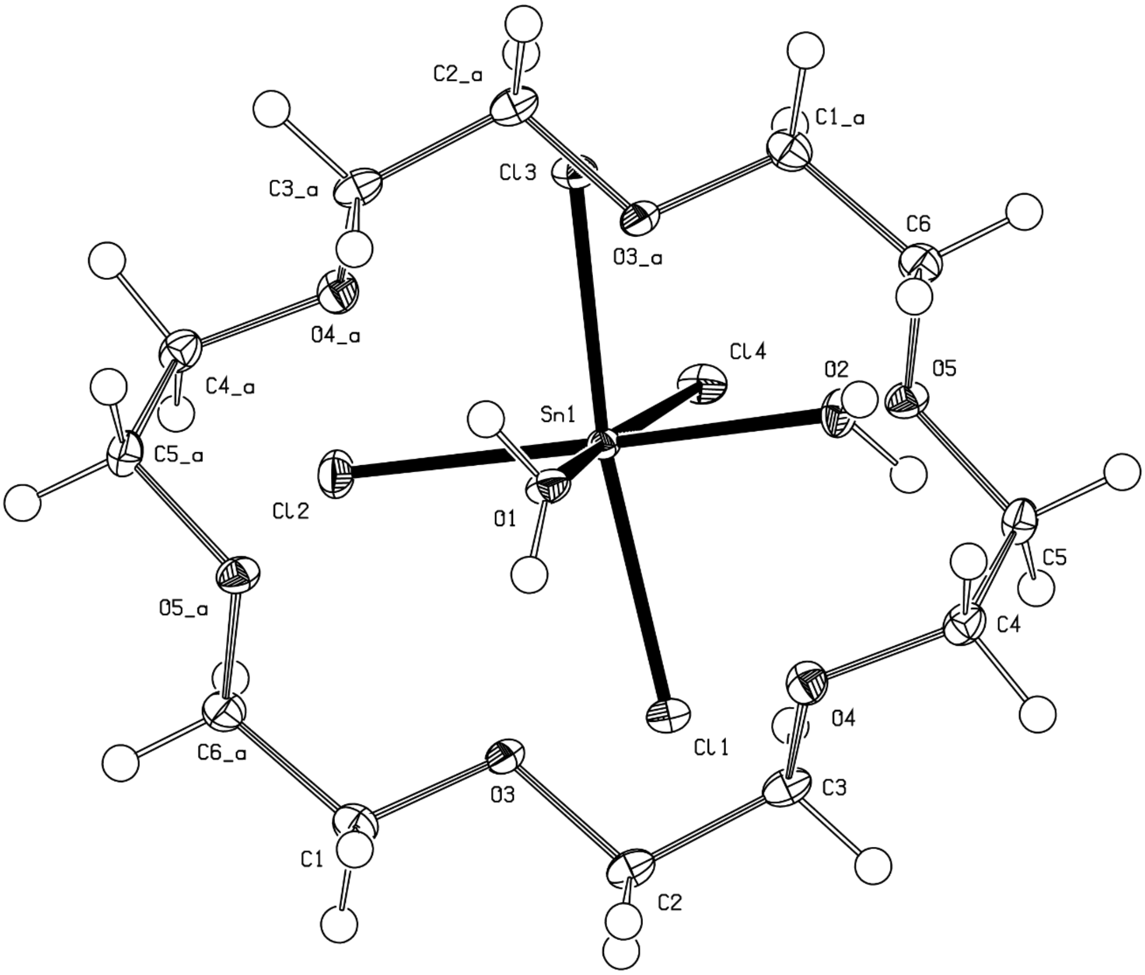

Supplementary Fig. 3. ORTEP representation of the asymmetric unit of **3**. Displacement ellipsoids are shown at 50% probability level.

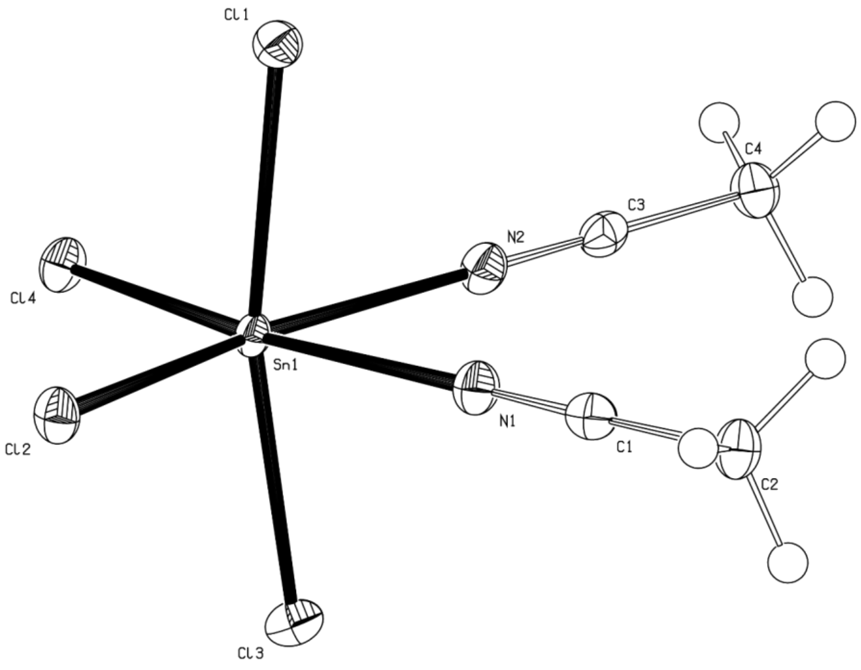

Supplementary Fig. 4. ORTEP representation of the asymmetric unit of **4**. Displacement ellipsoids are shown at 50% probability level.

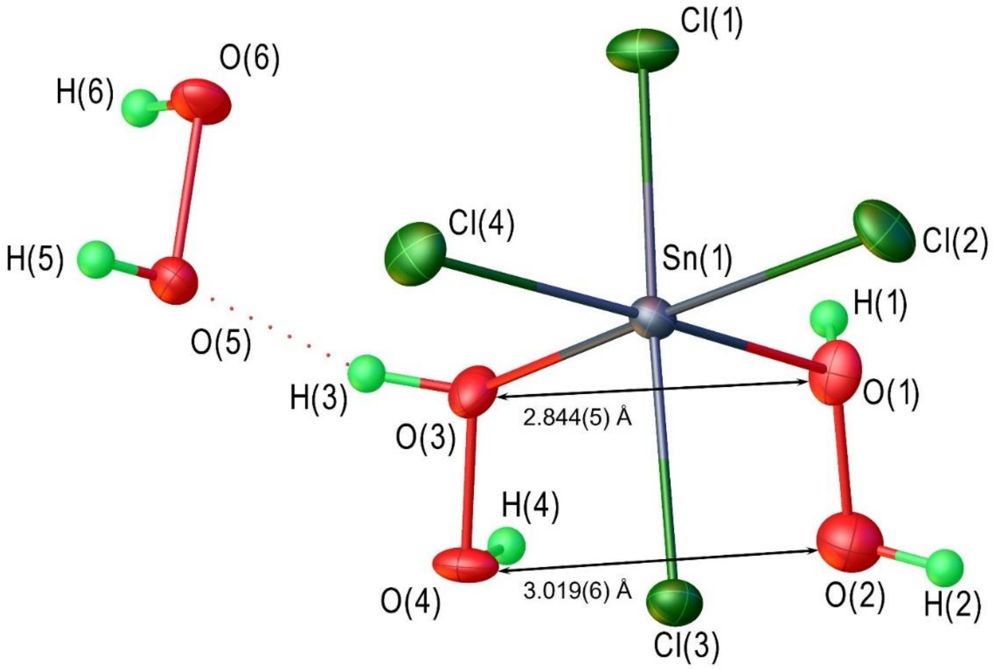

Supplementary Fig. 5. The O…O distances in $SnCl_4(H_2O_2)_2{\cdot}H_2O_2$ fragment in **1**. Crown ether is omitted for clarity.

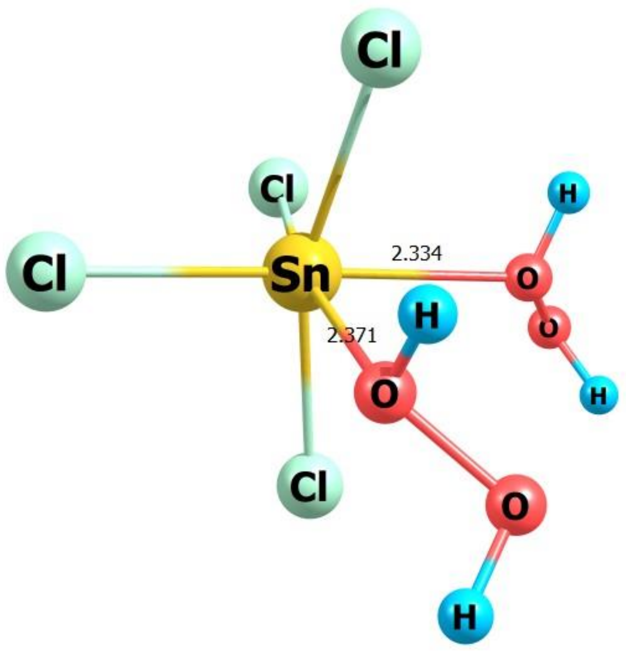

(a)

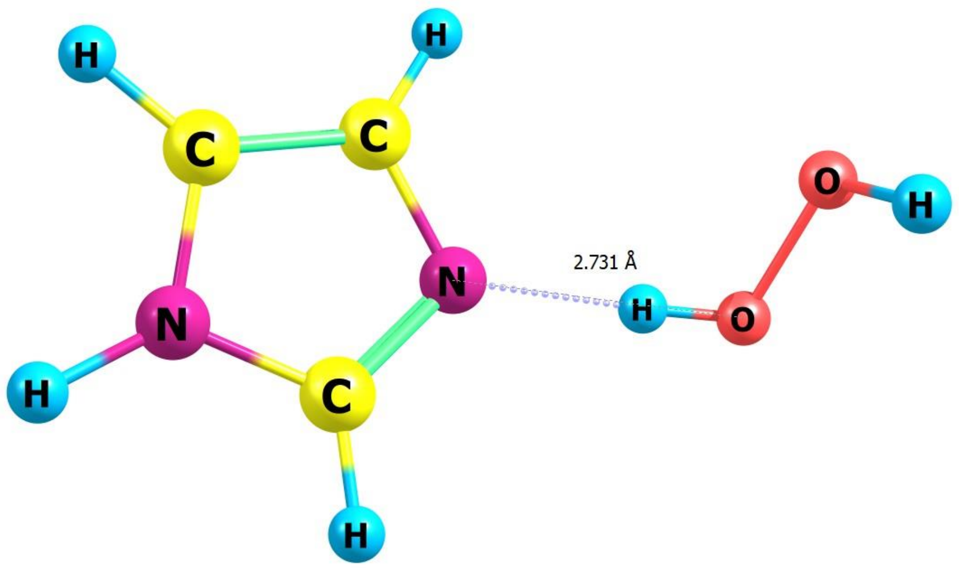

(b)

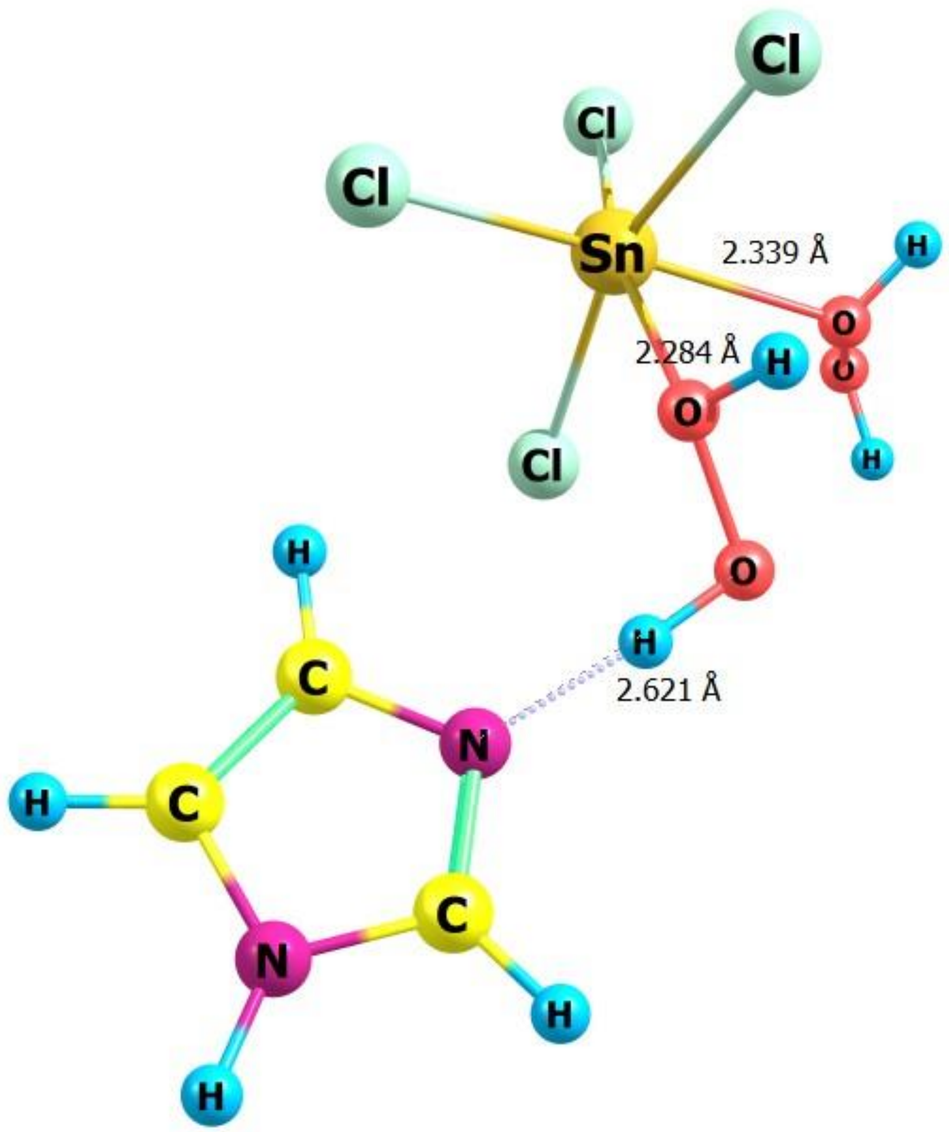
Cl
Cl
Cl
Sn
2.339 Å
H
O
2.284 Å
H
O
O
Cl
O
H
H
H
O
C
2.621 Å
N
H
C
C
N
H
H

(c)

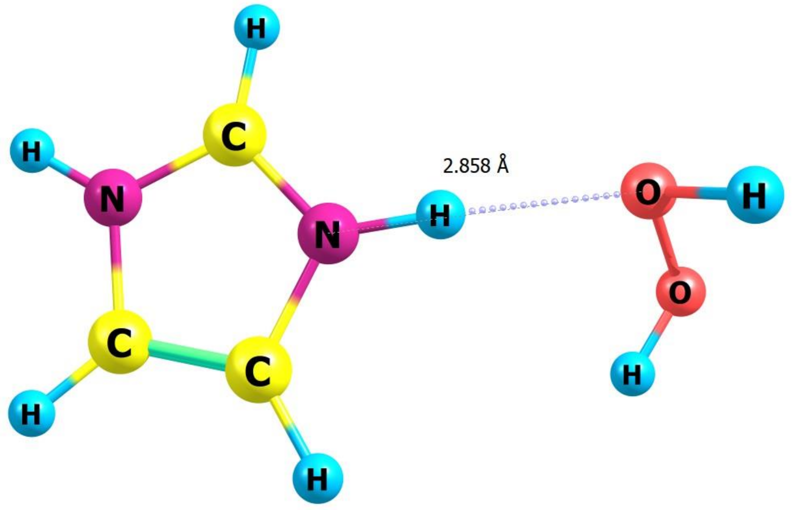
H
C
H
2.858 Å
N
N
H
O
H
O
C
C
H
H
H

(d)

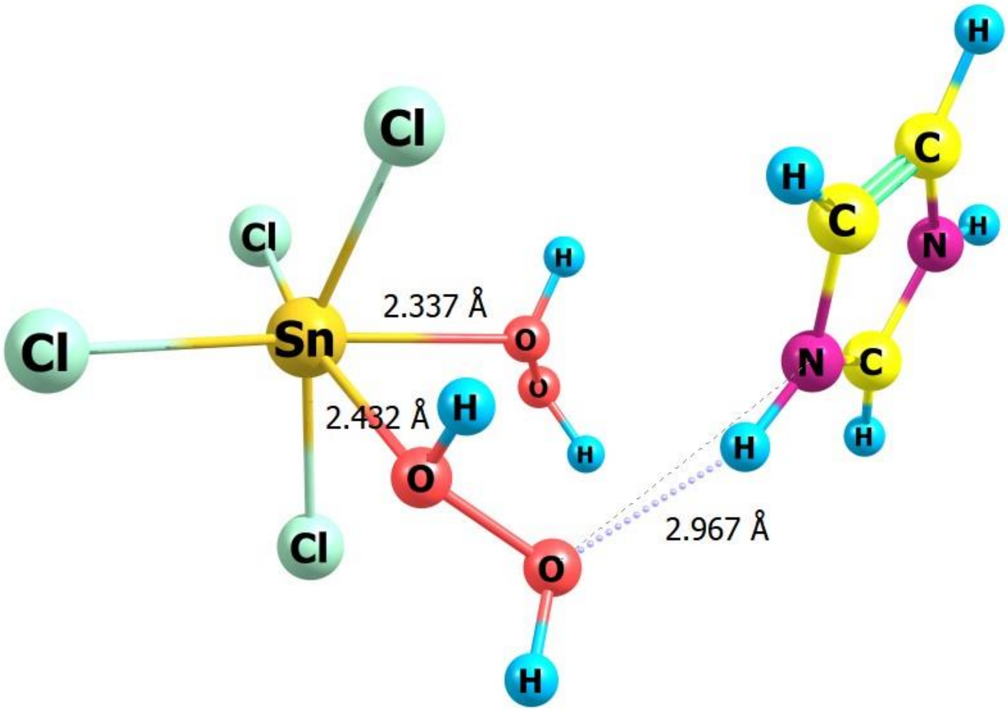

(e)

Supplementary Fig. 6. The optimized structures $SnCl_4(H_2O_2)_2$ (a), $H_2O_2{\cdot}C_3H_4N_2$ (b), $SnCl_4(H_2O_2)_2{\cdot}C_3H_4N_2$ (c), $H_2O_2{\cdot}C_3H_5N_2^+$ (d) and $SnCl_4(H_2O_2)_2{\cdot}C_3H_5N_2^+$ (e) at the ωB97X-D3/TZVPP level of theory.

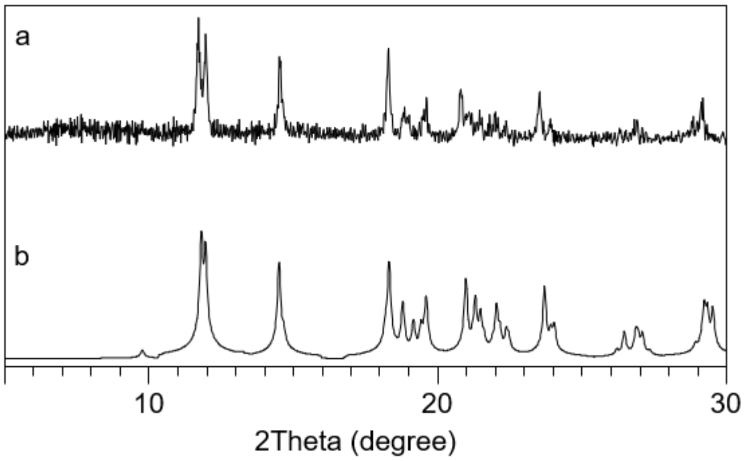

Supplementary Fig. 7. Experimental (a) and theoretical (b) powder XRD diffractograms of compound **3**.

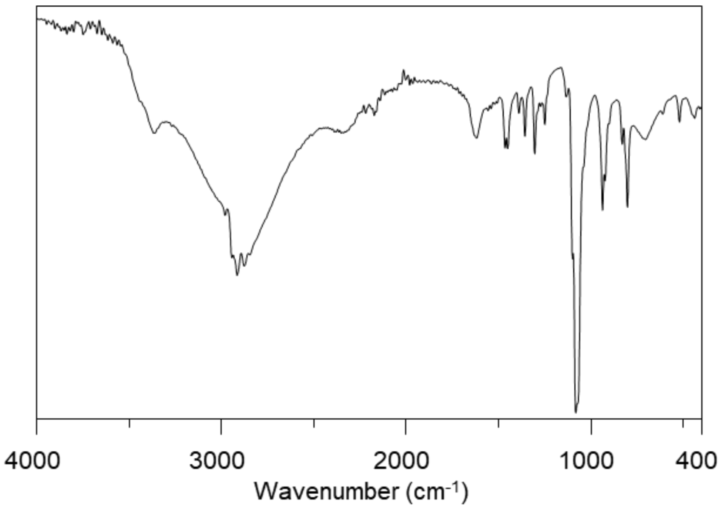

Supplementary Fig. 8. The FTIR spectrum of compound **3**.

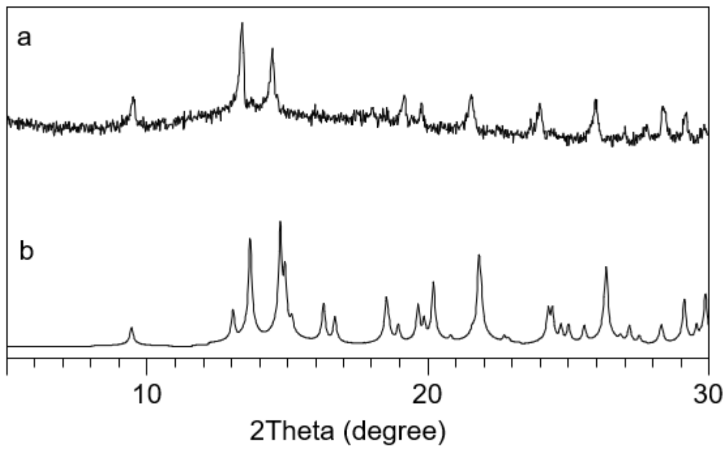

Supplementary Fig. 9. Experimental (a) and theoretical (b) powder XRD diffractograms of compound **4**.

Supplementary Table 1. Crystal data and details of X-ray analysis of **1-4**.

	**1**	**2**	**3**	**4**
formula	$C_{12}H_{30}O_{12}Cl_4Sn_1$	$C_{12}H_{32}O_{12}Cl_8Sn_2$	$C_{12}H_{32}O_{10}Cl_8Sn_2$	$C_4H_6N_2Cl_4Sn_1$
*F*w	626.85	889.35	857.35	342.60
colour, habit	colourless, prism	colourless, prism	colourless, prism	colourless, needle
cryst size (mm)	0.02×0.02×0.01	0.04×0.02×0.01	0.10×0.10×0.05	0.10×0.05×0.01
T (K)	100	100	100	100
crystal system	orthorhombic	triclinic	monoclinic	monoclinic
space group	*Pbca*	*P*-1	$P2_1/c$	$P2_1/c$
a (Å)	15.945(2)	9.169(1)	9.391(1)	6.020(1)
b (Å)	16.039(2)	9.692(1)	18.107(1)	13.552(1)
c (Å)	18.256(1)	9.825(1)	9.442(1)	13.127(1)
α (deg)	90	90.898(2)	90	90
β (deg)	90	110.985(2)	118.867(2)	99.481(2)
γ (deg)	90	116.749(2)	90	90
V (Å^3)	4669.0(7)	711.35(8)	1406.11(14)	1056.34(11)
Z	8	1	2	4
D_c (g·cm^{-3})	1.784	2.076	2.025	2.154
μ (mm^{-1})	1.605	2.557	2.578	3.374
F(000)	2528	436	840	648
θ range (deg)	2.54 to 28.14	2.27 to 30.44	2.71 to 30.54	3.01 to 30.49
refl collcd	18120	5424	14125	10704
indep reflns / R_{int}	4477 / 0.081	2753 / 0.026	3724 / 0.042	2542/0.029
reflns $I>2\sigma(I)$	3024	2416	3137	2192
No of param	274	169	161	102
GooF on F^2	1.021	1.094	1.035	1.110
R_1 ($I>2\sigma(I)$)	0.0438	0.0347	0.0280	0.0218
wR_2(all data)	0.0867	0.0621	0.0607	0.0445
largest diff peak / hole (e·Å^{-3})	0.51 / -0.59	0.70 / -0.78	0.64 / -1.00	0.78/-0.63
**CCDC number**	**2260843**	**2260844**	**2260845**	**2260846**

Supplementary Table 2. Selected geometric parameters of **1-3.**

Parameter	**1**	**2**	**3**
d(Sn-O_2H_2), Å	2.179(4)<br>2.200(3)	2.225(3)	
d(Sn-OH_2), Å		2.137(3)	2.133(2)<br>2.138(2)
∠(O-Sn-O), °	81.02(14)	77.16(12)	83.76(8)
d(O-O), Å<br>O(1)-O(2)<br>O(3)-O(4)<br>O(5)-O(6)	 1.422(5)<br>1.443(5)<br>1.469(5)	 1.445(4)<br>-<br>-	 -<br>-<br>-
d(O…O), Å<br>O(1)…O(3)<br>O(2)…O(4)<br>O(1)…O(2)	 2.844(5)<br>3.019(6) 	 2.722(4) 	 <br><br>2.851(3)

Supplementary Table 3. Geometric parameters of hydrogen bonds in the structure **1**.

D–H…A	D–H, Å	H…A, Å	D…A, Å	∠ D–H…A, °
O(1)-H(1)…O(8)	0.85(3)	1.74(3)	2.583(5)	168(6)
O(2)-H(2)…O(10)	0.86(3)	1.82(3)	2.671(5)	167(5)
O(3)-H(3)…O(5)	0.85(3)	1.74(3)	2.542(5)	158(5)
O(4)-H(4)…O(7)	0.85(3)	1.91(3)	2.730(5)	162(3)
O(5)-H(5)…O(12)^{i}	0.84(3)	2.00(3)	2.823(5)	166(3)
O(6)-H(6)…O(9)^{i}	0.85(3)	1.99(3)	2.825(5)	166(4)

Symmetry operation: (i) x+1/2, y, −z+3/2.

Supplementary Table 4. Geometric parameters of hydrogen bonds in the structure **2**.

D–H…A	D–H, Å	H…A, Å	D…A, Å	∠ D–H…A, °
O(1)-H(1)…O(6)	0.86(2)	1.70(3)	2.548(4)	168(8)
O(2)-H(2)…Cl(3)^{i}	0.88(2)	2.33(4)	3.114(4)	149(7)
O(3)-H(31)…O(4)	0.84(2)	1.86(4)	2.664(4)	161(5)
O(3)-H(32)…O(5)^{ii}	0.85(2)	1.79(4)	2.640(4)	173(6)

Symmetry operations: (i) -x, -y, -z; (ii) -x+1, -y+1, -z+1.

Supplementary Table 5. Geometric parameters of hydrogen bonds in the structure **3**.

D–H…A	D–H, Å	H…A, Å	D…A, Å	∠ D–H…A, °
O(1)-H(11)…O(3)	0.83(2)	1.88(2)	2.696(3)	168(4)
O(1)-H(12)…O(4)[i]	0.83(2)	1.81(2)	2.628(3)	172(4)
O(2)-H(21)…O(5)	0.85(2)	1.77(2)	2.614(3)	172(3)
O(2)-H(22)…Cl(4)[ii]	0.84(2)	2.39(2)	3.215(2)	170(3)

Symmetry operations: (i) -x+1, -y+1, -z+1; (ii) x+2, -y+0.5, z+0.5.

Supplementary Table 6. Selected properties of the electron density at bond critical points according to Bader analysis.

**Compound**	**Contact**	**ρ**	**∇ρ**	**$\nabla^2\rho$**	**Gb**	**Vb**
**1**[a]	Sn(1)-O(1)	0.0610	2.95E-15	0.1899	0.0609	-0.0744
	Sn(1)-O(3)	0.0531	3.07E-17	0.1573	0.0500	-0.0608
	H(1)…O(8)	0.0462	5.14E-15	0.1017	0.0359	-0.0464
	H(2)…O(10)	0.0388	2.22E-16	0.0928	0.0302	-0.0371
	H(3)…O(5)	0.0443	5.51E-14	0.0907	0.0329	-0.0431
	H(4)…O(7)	0.0088	7.01E-18	0.0371	0.0075	-0.0057
	H(4)…O(12)	0.0256	7.55E-15	0.0775	0.0204	-0.0214
**2**[b]	Sn(1)-O(1)	0.0490	3.21E-17	0.1463	0.0458	-0.0550
	Sn(1)-O(3)	0.0746	8.11E-17	0.2643	0.0833	-0.1004
	H(1)…O(6)	0.0443	8.57E-17	0.1045	0.0351	-0.0440
	H(2)…Cl(4)	0.0230	1.83E-16	0.0568	0.0150	-0.0158
	H(31)…O(4)	0.0484	9.68E-16	0.0983	0.0366	-0.0485
	H(32)…O(5)	0.0421	3.46E-15	0.0943	0.0321	-0.0406
$SnCl_4(H_2O_2)_2$ [c]	Sn(1)-O(1)	0.0477	2.32E-14	0.1401	0.0437	-0.0523
	Sn(1)–O(3)	0.0518	5.29E-15	0.1549	0.0489	-0.0590
$H_2O_2{\cdot}C_3H_4N_2$	N…H	0.0500	7.14E-17	0.0710	0.0309	-0.0441
$SnCl_4(H_2O_2)_2{\cdot}C_3H_4N_2$	Sn(1)-O(1)	0.0582	9.04E-15	0.1749	0.0564	-0.0691
	Sn(1)–O(3)	0.0511	8.88E-15	0.1531	0.0482	-0.0580
	N…H	0.0699	3.48E-16	0.0558	0.0405	-0.0670
$H_2O_2{\cdot}C_3H_5N_2^+$	H…O	0.0330	3.42E-13	0.0902	0.0259	-0.0293
$SnCl_4(H_2O_2)_2{\cdot}C_3H_5N_2^+$	Sn(1)-O(1)	0.0415	6.88E-17	0.1220	0.0368	-0.0430
	Sn(1)–O(3)	0.0512	9.25E-12	0.1540	0.0483	-0.0582
	H…O	0.0217	4.28E-16	0.0754	0.0181	-0.0173

[a] See Figure 2A for atom labeling; [b] See Figure 2B for atom labeling, [c] See Table 2 for atom labeling.

Supplementary Table 7. FTIR band assignment of **3**.

Wavenumber, cm^{-1}	Assignment	Reference
3439 w (shoulder)	ν(O–H) in H_2O	2, 20
3365 m, br	ν(O–H) in H_2O	2, 20
2983-2848 s, sh	ν_s(C–H)/ν_{as}(C–H) in 18-crown-6	2
1621 w, br	δ(H–O–H)	2, 20
1490-1136 w, sh	δ(CH_2) and ρ(CH_2) in 18-crown-6	21
1084 vs, sh	ν_{as}(C-O-C) in 18-crown-6	2, 20, 21
970-780 m, sh	ρ(CH_2) in 18-crown-6	21
720 w, br	ρ_r(H_2O)	22, 23, 24
614 w, br	ρ_w(H_2O)	22, 23, 24
540-442 w, br	ν(Sn–O)	25

Supplementary References

1. Schumb, W. C., Satterfield, C. N. & Wentworth, R. L. *Hydrogen peroxide*. (Reinhold Publishing Corporation, 1955).

2. Antsyshkina, A. S. et al. Synthesis and structure of tin tetrachloride adducts with crown ether: Crystal structure of $[Sn(H_2O)_2Cl_4]\cdot18C6$ and $[Sn(H_2O)_2Cl_4]\cdot18C6\cdot2H_2O$. *Russ. J. Inorg. Chem.* **56**, 530–538 (2011).

3. Charlot, G. Les méthodes de la chimie analytique: analyse quantitative minérale. (Masson, 1966).

4. Neese, F. The ORCA program system. *WIREs Comput. Mol. Sci.* **2**, 73–78 (2012).

5. Neese, F. Software update: The ORCA program system—Version 5.0. *WIREs Comput. Mol. Sci.* **12**, (2022).

6. van Wüllen, C. Molecular density functional calculations in the regular relativistic approximation: Method, application to coinage metal diatomics, hydrides, fluorides and chlorides, and comparison with first-order relativistic calculations. *J. Chem. Phys.* **109**, 392–399 (1998).

7. Lin, Y.-S., Li, G.-D., Mao, S.-P. & Chai, J.-D. Long-Range Corrected Hybrid Density Functionals with Improved Dispersion Corrections. *J. Chem. Theory Comput.* **9**, 263–272 (2013).

8. Grimme, S., Antony, J., Ehrlich, S. & Krieg, H. A consistent and accurate ab initio parametrization of density functional dispersion correction (DFT-D) for the 94 elements H-Pu. *J. Chem. Phys.* **132**, 154104 (2010).

9. Rolfes, J. D., Neese, F. & Pantazis, D. A. All-electron scalar relativistic basis sets for the elements Rb–Xe. *J. Comput. Chem.* **41**, 1842–1849 (2020).

10. Barone, V. & Cossi, M. Quantum Calculation of Molecular Energies and Energy Gradients in Solution by a Conductor Solvent Model. *J. Phys. Chem. A* **102**, 1995–2001 (1998).

11. Frisch, M. J. *et al.* Gaussian 09, Rev. D.01. *Gaussian Inc., Wallingford CT* Wallingford CT (2009) doi:10.1159/000348293.

12. Chai, J.-D. & Head-Gordon, M. Long-range corrected hybrid density functionals with damped atom–atom dispersion corrections. *Phys. Chem. Chem. Phys.* **10**, 6615 (2008).

13. Becke, A. D. Density-functional thermochemistry. III. The role of exact exchange. *J. Chem. Phys.* **98**, 5648–5652 (1993).

14. Lee, C., Yang, W. & Parr, R. G. Development of the Colle-Salvetti correlation-energy formula into a functional of the electron density. *Phys. Rev. B* **37**, 785–789 (1988).

15. Perdew, J. P., Burke, K. & Ernzerhof, M. Generalized Gradient Approximation Made Simple. *Phys. Rev. Lett.* **77**, 3865–3868 (1996).

16. Weigend, F. & Ahlrichs, R. Balanced basis sets of split valence, triple zeta valence and quadruple zeta valence quality for H to Rn: Design and assessment of accuracy. *Phys. Chem. Chem. Phys.* **7**, 3297 (2005).

17. Metz, B., Stoll, H. & Dolg, M. Small-core multiconfiguration-Dirac–Hartree–Fock-adjusted pseudopotentials for post- d main group elements: Application to PbH and PbO. *J. Chem. Phys.* **113**, 2563–2569 (2000).

18. Bader, R. F. W. *Atoms in Molecules: A Quantum Theory*. (Oxford University Press, 1990).

19. Keith, T. A. AIMALL. (2019).

20. Junk, P. C. & Raston, C. L. Hydrolytic stability of $SnCl_4$ and $GaCl_3$ in the formation of [*cis*-SnCl4(H_2O)$_2$]·18-crown-6·2H_2O and [[2,2,2]cryptand+2H^+][$GaCl_4$]$_2$. *Inorganica Chim. Acta* **357**, 595–599 (2004).

21. Bühl, M., Ludwig, R., Schurhammer, R. & Wipff, G. Hydronium Ion Complex of 18-Crown-6: Theory Confirms Three "Normal" Linear Hydrogen Bonds. *J. Phys. Chem. A* **108**, 11463–11468 (2004).

22. Manna, S. C., Mistri, S. & Jana, A. D. A rare supramolecular assembly involving ion pairs of coordination complexes with a host–guest relationship: synthesis, crystal structure, photoluminescence and thermal study. *CrystEngComm* **14**, 7415 (2012).

23. Hetmańczyk, J. & Hetmańczyk, Ł. Vibrational and reorientational dynamics and thermal properties in [Mg(H_2O)$_4$](ReO_4)$_2$ supported by periodic DFT study. *Vib. Spectrosc.* **94**, 49–60 (2018).

24. Fujita, J., Nakamoto, K. & Kobayashi, M. Infrared Spectra of Metallic Complexes. II. The Absorption Bands of Coördinated Water in Aquo Complexes. *J. Am. Chem. Soc.* **78**, 3963–3965 (1956).

25. Shihada, A.-F., Abushamleh, A. S. & Weller, F. Crystal Structures and Raman Spectra of *cis*-[$SnCl_4$(H_2O)$_2$]·2H_2O, *cis*-[$SnCl_4$(H_2O)$_2$]·3H_2O, [Sn_2Cl_6(OH)$_2$(H_2O)$_2$]·4H_2O, and [HL][$SnCl_5$(H_2O)]·2.5H_2O (L=3-acetyl-5-benzyl-1-phenyl-4, 5-dihydro-1, 2, 4-triazine-6-one oxime, $C_{18}H_{18}N_4O_2$). *Z. Anorg. Allg. Chem.* **630**, 841–847 (2004).
