# Supplementary material for: Synergism of primary and secondary interactions in a crystalline hydrogen peroxide complex with tin
Source: Nat Commun. 2024 Jul 9;15:5758. doi: 10.1038/s41467-024-50164-9 (PMC11233698; doi:10.1038/s41467-024-50164-9)
Supplement: Supplementary file 3 — Description of Additional Supplementary Files [file 41467_2024_50164_MOESM3_ESM.pdf]

Description of Additional Supplementary Files

File name: Supplementary Data 1
Description: The atomic Cartesian coordinates of the optimized computational models.

File name: Supplementary Movie 1
Description: A biphasic mixture formed by the careful addition of a fourfold molar excess of anhydrous hydrogen peroxide to tin(IV) chloride.
